# Characteristics of cerebral glucose metabolism in patients with cognitive impairment in multiple system atrophy

**DOI:** 10.3389/fnagi.2025.1520515

**Published:** 2025-03-05

**Authors:** Bin Chen, Lingchao Li, Lin Bai, Min Zhao, Ying Chang, Shi Gao

**Affiliations:** China-Japan Union Hospital, Jilin University, Changchun, China

**Keywords:** multiple system atrophy, cognitive impairment, ^18^F-fluorodeoxyglucose, positron emission tomography, brain metabolism

## Abstract

**Objective:**

We aimed to conduct ^18^F-fluorodeoxyglucose (^18^F-FDG) positron emission tomography (PET) to investigate the metabolic changes in brain regions associated with cognitive decline in patients with multiple system atrophy (MSA) and to assess the diagnostic efficacy of ^18^F-FDG PET imaging for evaluating the cognitive status of MSA patients.

**Methods:**

This study included 44 MSA patients (MSA group) and 30 healthy controls (HC group) who underwent brain ^18^F-FDG PET imaging. All patients were subjected to the Mini-Mental State Examination and categorized into the MSA with normal cognition (MSA-NC) and MSA with cognitive impairment (MSA-CI) groups. Statistical parametric mapping (version 12) was used to analyze PET images and compare the differences in brain metabolism between the MSA and HC groups. The PET images of MSA-CI and MSA-NC patients were compared to analyze the metabolic characteristics, and the regional cerebral metabolic rate of glucose (rCMRglc) was calculated for different brain regions. Receiver operating characteristic (ROC) curves were used to analyze the ability of the rCMRglc of different brain regions to assess the cognitive status of MSA patients.

**Results:**

Compared with the HC group, the MSA group showed diffuse reductions in glucose metabolism in the cerebellar regions, decreased metabolism in specific areas of the left inferior parietal lobule, right putamen, and left middle temporal gyrus, and increased metabolism in the left postcentral gyrus, right postcentral gyrus, left precuneus. Compared with the MSA-NC group, the MSA-CI group exhibited decreased metabolism in the right superior frontal gyrus and right superior parietal lobule. The rCMRglc value of the right superior frontal gyrus (Montreal Neurological Institute coordinates: 18, −6, 70) showed better diagnostic efficacy for identifying MSA-CI, with an area under the ROC curve of 0.829 (95%CI = 0.696–0.963), sensitivity of 84.6% (95%CI = 66.5–93.9%), and specificity of 83.3% (95%CI = 60.8–94.2%).

**Conclusion:**

Compared with MSA-NC patients, the MSA-CI patients show decreased metabolism in the right superior frontal gyrus and right superior parietal lobule. The rCMRglc value of the right superior frontal gyrus may be a potential molecular imaging biomarker for diagnosing MSA-CI.

## Introduction

Multiple system atrophy (MSA) is a rare and fatal neurodegenerative disease characterized by Parkinsonism, cerebellar damage, and autonomic dysfunction (Stefanova et al., 2009; Wenning et al., 2022). MSA can be further classified in a parkinsonian-type MSA (MSA-P), if Parkinsonism is the uppermost feature, and in a cerebellar-type MSA (MSA-C) if the cerebellar syndrome prevails (Niccolini and Politis, 2016; Goolla et al., 2023). Previous studies did not consider cognitive impairment to be present in MSA patients and included it as an exclusion criterion (Gilman et al., 2008). However, progressing research has gradually recognized that some MSA patients exhibit cognitive impairment (Brown et al., 2010). In 2014, the Movement Disorder Society stated that cognitive impairment may be an under-recognized clinical feature of MSA (Stankovic et al., 2014). Cognitive impairment in MSA patients manifests as broad deficits across multiple cognitive domains, including executive function, attention, visuospatial abilities, and memory (Brown et al., 2010; Koga et al., 2017; Watanabe et al., 2023). Given its insidious onset and atypical symptoms, it is challenging to diagnose the disease before death. In clinical practice, doctors primarily rely on clinical manifestations and related scales to determine whether patients have cognitive impairment (Folstein et al., 1975; Mitchell, 2009; Stankovic et al., 2014). However, these scales have significant subjectivity, require physicians with specialized training and experience, have poor reproducibility, and do not reflect the patients condition fully, rendering them inadequate as the sole basis for a definitive diagnosis.

Currently, some diagnostic methods have been developed to assist in diagnosing cognitive impairment in MSA. Regarding body fluid testing, the biomarker detection in blood urine and cerebrospinal fluid testing samples remains in the research phase and is prone to errors and biases at various stages from extraction to detection, which limits those use (Laurens et al., 2015; Cong et al., 2021). In structural imaging, a series of morphological magnetic resonance imaging (MRI) studies have shown that brain atrophy in MSA patients occurs in widespread cortical and subcortical structures, including the striatum, midbrain, thalamus, and cerebellum. However, some conclusions remain controversial and require further exploration (Kim et al., 2015; Lee et al., 2016; Caso et al., 2020; Cao et al., 2021). The pathological changes in brain function and metabolism often precede structural changes such as brain atrophy. Therefore, highly sensitive and specific biomarkers or imaging markers to improve diagnostic reliability are urgently needed. ^18^F-fluorodeoxyglucose (^18^F-FDG) positron emission tomography (PET) is a commonly used imaging method for neurodegenerative diseases. Literature has reported the potential application of ^18^F-FDG PET in evaluating cognitive impairment in Parkinson disease (Garcia-Garcia et al., 2012; Booth et al., 2022). Thus, although existing research is limited, ^18^F-FDG PET can be used to investigate cognitive impairment in MSA. This study aimed to investigate the cognitive profile of MSA patients, validate the characteristics of brain glucose metabolism in MSA patients, detect regional brain glucose metabolic differences between MSA patients with different cognitive statuses, and explore the utility of brain glucose metabolism in differentiating the cognitive status in MSA.

## Materials and methods

### Patient data

The study included 44 patients with MSA, who were treated at the Department of Neurology, China-Japan Union Hospital, Jilin University between June 2020 and March 2023. The clinical diagnosis of MSA for all patients was made by senior neurologists based on the 2022 clinical diagnostic criteria for MSA proposed by the International Parkinson and Movement Disorder Society (Wenning et al., 2022). The exclusion criteria were as follows: (1) presence of severe or unstable diseases in the late stages that could affect the evaluation of the scale results; (2) a history of acute cerebrovascular disease within the last 3 months; (3) currently experiencing active epilepsy; (4) a history of psychosis; and (5) a history of dementia due to other known causes, such as poisoning. Demographic information and clinical data were collected for each participant, including sex, age, education level, disease duration, and the Unified Parkinson Disease Rating Scale-III (UPDRS-III). Cognitive function was assessed using the Mini-Mental State Examination (MMSE), which was jointly completed by neurologists and neuroimaging physicians who had undergone professional training. The time interval between performing the MMSE and ^18^F-FDG PET was set to be within 1 week. The study also included a healthy control (HC) group comprising 30 individuals, meticulously matched for sex, age, education level, and geographic region, and had no history of neurological or psychiatric illnesses, had normal neurological examination results, and had a MMSE score of ≥26. Based on the diagnostic criteria for Parkinson disease-related cognitive impairment (Emre et al., 2007; Litvan et al., 2012), the MSA patients were categorized into the MSA with normal cognition (MSA-NC) and MSA with cognitive impairment (MSA-CI) groups. Both MSA and HC group patients were right-handed. All participants provided written informed consent before enrolling in the study.

### ^18^F-FDG PET imaging

The ^18^F-FDG injection was prepared by the PET Center of the Nuclear Medicine Department, China-Japan Union Hospital, Jilin University. Patients were instructed to avoid using levodopa and other adjunctive medications that could affect cerebral glucose metabolism and fast for over 6 h before the scheduled imaging. Fasting blood glucose levels were required to be below 10 mmol/L. The ^18^F-FDG (0.1 mCi/kg) was injected through the cubital vein. After injection, patients were allowed to rest in a quiet, light-protected environment for 60 min before undergoing head PET imaging.

The imaging was performed using the uMI 780 scanner (United Imaging Healthcare, Shanghai, China). A computed tomography (CT) scan was first conducted, covering the area from the top of the head to the lower cerebellum, with the following parameters: tube voltage, 120 kV; tube current, 320 mAs; rotation time, 0.8 s; pitch, 0.675 mm; slice thickness; 3.0 mm; and scanning matrix, 512 × 512. Subsequently, a head PET scan was performed in the three-dimensional (3D) mode for the same area, with the following parameters: scanning duration, 10 min; slice thickness, 1.34 mm; and scanning matrix, 256 × 256. Images were reconstructed using the ordered-subset expectation maximization algorithm with 2 iterations and 20 subsets. Additionally, the PET images were corrected for attenuation using the CT scan data.

### PET image analysis

All original DICOM images were converted into analyzable NIfTI format using MRIcron.^1^ On the MATLAB R2022a platform (MathWorks Inc., Sherborn, MA, United States), the statistical parametric mapping (SPM) version 12 analysis software^2^ was employed for voxel-wise image analysis. First, the PET images were standardized according to the Montreal Neurological Institute (MNI) brain atlas, transforming them into images conforming to a standard anatomical space. Following this, a 2 × 2 × 2 mm voxel was constructed, and the normalized images were smoothed with isotropic 3D Gaussian kernel (full width at half maximum = 8 mm) to increase the signal-to-noise ratio.

After preprocessing, SPM12 software was used to analyze the differences in brain glucose metabolism between the MSA and HC groups and compare the variations in brain glucose metabolism among MSA patients with different cognitive statuses. Age, sex, education level, and UPDRS-III scores were included as covariates in the statistical analyses to exclude their influence on the results. The preprocessed PET image values were adjusted to a mean value of 50 mL/dL/min by “proportional scaling” to reduce individual variation. A mask with a 0.8 intensity value was used to select voxel activity and exclude extracranial activities.

### Extraction and calculation of the regional cerebral glucose metabolic rate of glucose

The regions of interest were delineated in the brain areas where significant differences were observed between the MSA-CI and MSA-NC groups. MARSBAR software^3^ was used to quantify the glucose metabolism values within each volume of interest (VOI) and the whole brain glucose metabolism value. The regional cerebral glucose metabolic rate of glucose (rCMRglc) was calculated using the following formula:


$$\begin{array}{l} \mathrm{rCMRglc}=\left(VOI\;\mathrm{value}/\mathrm{whole brain glucose metabolism value}\right)\\ \;\times 50\times 100\%\text{.} \end{array}$$

This measure was used for subsequent analyses of glucose metabolism differences among MSA patients with varying cognitive statuses and for constructing receiver operating characteristic (ROC) curves. The SPM results were registered to a standard brain T1-weighted MRI using MRIcron software. A significance level of *p*-value <0.01 (uncorrected) or after false discovery rate correction was applied.

### Statistical analysis

In the statistical analysis of patient data, quantitative data were subject to normality assessments. The variables following a normal distribution are presented as means ± standard deviations, while those not conforming to this distribution are presented as medians (interquartile ranges). The *t*-test was used to compare continuous data with a normal distribution, while the Mann–Whitney *U* test was used for continuous data exhibiting non-normal distribution. The Pearson *χ*^2^ test was applied for categorical count data. ROC curve analysis was utilized to evaluate the capacity of the rCMRglc of different brain regions in assessing the cognitive impairment of patients and identifying the optimal cutoff values. All statistical analyses were performed using Statistical Product and Service Solutions, version 24.0 (IBM Corporation, Armonk, NY, United States). Two-tailed *p*-values <0.05 were considered statistically significant.

## Results

### General data

This study included 44 patients diagnosed with MSA, comprising 30 patients with MSA-C and 14 patients with MSA-P. There were 18 males and 26 females, with an average age of 58.8 ± 7.4 years. The median education level was 6 years, the median disease duration was 2 years, and the mean UPDRS-III score was 38.5 ± 18.2. The HC group included 30 individuals, exhibiting no statistically significant differences in age or education level when compared with the MSA group. The median MMSE score of the MSA group was significantly lower than that of the HC group (26 vs. 30; *p* < 0.001) (Table 1).

**Table 1:** Demographic characteristics of the multiple system atrophy and healthy control groups.

	MSA group (*n* = 44)	HC group (*n* = 30)	*p*-value
Age (years)	59.7 ± 8.3	58.8 ± 7.4	0.654
Sex (male/female)	18/26	10/20	0.509
Education level (years)	9 (6, 12)	9 (6, 11)	0.700
MMSE score	26 (23, 27)	30 (30, 30)	<0.001
MSA duration (years)	2.0 (1.0, 3.8)	/	/
UPDRS-III score	38.5 ± 18.2	/	/

All data are presented as numbers, means ± standard deviations, or medians (interquartile ranges). MSA, multiple system atrophy; HC, healthy control; MMSE, Mini-Mental State Examination; UPDRS-III, Unified Parkinson Disease Rating Scale.

Among the 44 MSA patients, 26 were categorized as MSA-NC and 18 as MSA-CI. No statistically significant differences were observed in age, education level, disease duration, and UPDRS-III scores between the MSA-NC and MSA-CI groups. The median MMSE score was significantly higher in the MSA-NC group than in the MSA-CI group (27 vs. 24; *p* < 0.001) (Table 2).

**Table 2:** Demographic characteristics of the multiple system atrophy patients with normal cognition and with cognitive impairment.

	MSA-NC group (*n* = 26)	MSA-CI group (*n* = 18)	*p*-value
Age (years)	58.7 ± 9.3	61.1 ± 6.5	0.347
Sex (male/female)	12/14	6/12	0.395
Education level (years)	9 (7, 12)	8 (6, 10)	0.076
MMSE score	27 (26, 29)	24 (23, 24)	<0.001
MSA duration (years)	2.0 (1.0, 2.3)	2.0 (0.9, 4.5)	0.089
UPDRS-III score	35.8 ± 18.4	42.5 ± 17.8	0.233

All data are presented as numbers, means ± standard deviations, or medians (interquartile ranges). MSA-NC, multiple system atrophy with normal cognition; MSA-CI, multiple system atrophy with cognitive impairment; MSA, multiple system atrophy; MMSE, Mini-Mental State Examination; UPDRS-III, Unified Parkinson Disease Rating Scale.

### Differences in the brain glucose metabolism between the MSA and HC groups

**Figure 1:**
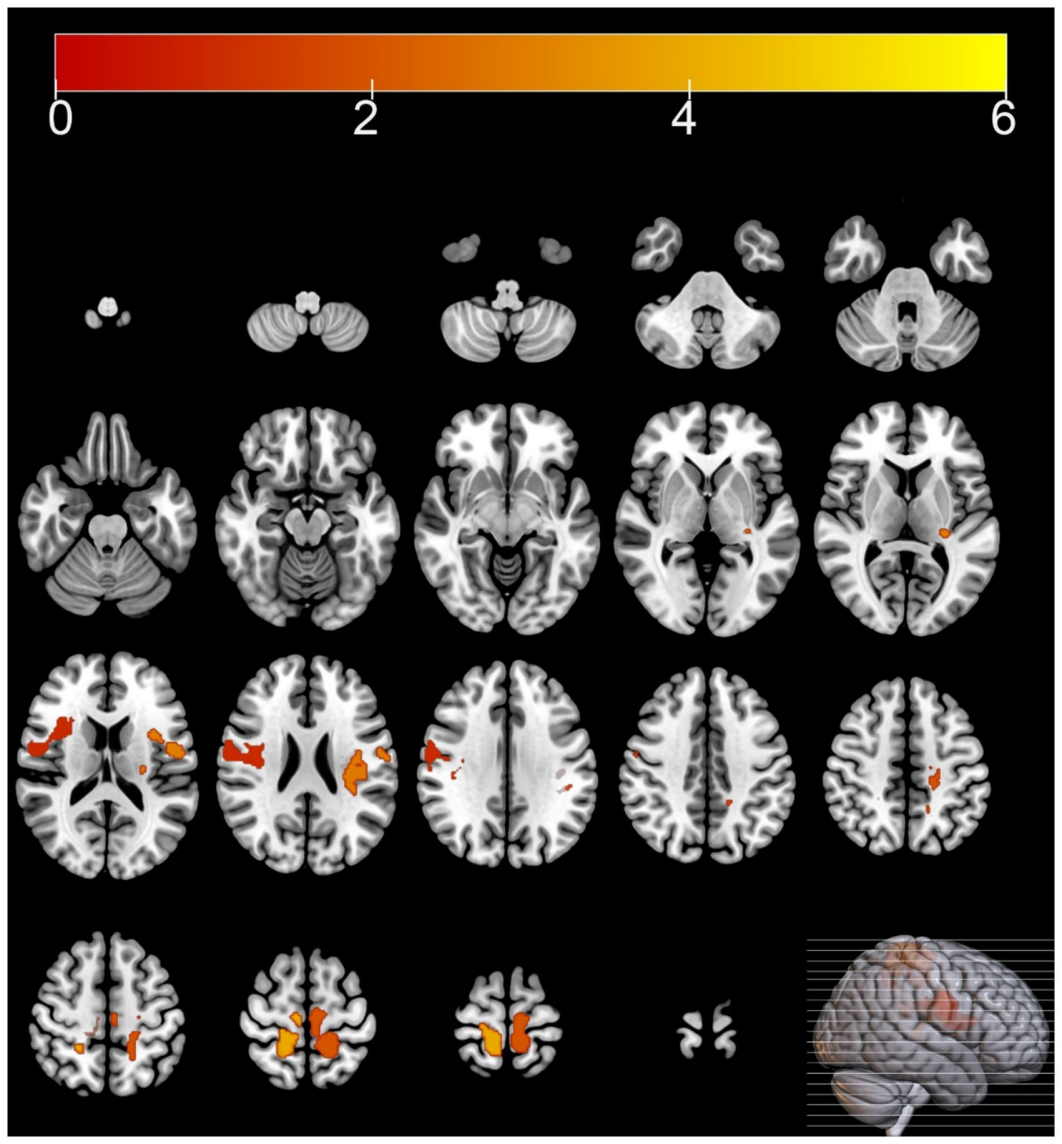
Brain regions with hypermetabolism differences between the multiple system atrophy and healthy control groups. The thresholds of the color bars depict the *T*-values.

**Figure 2:**
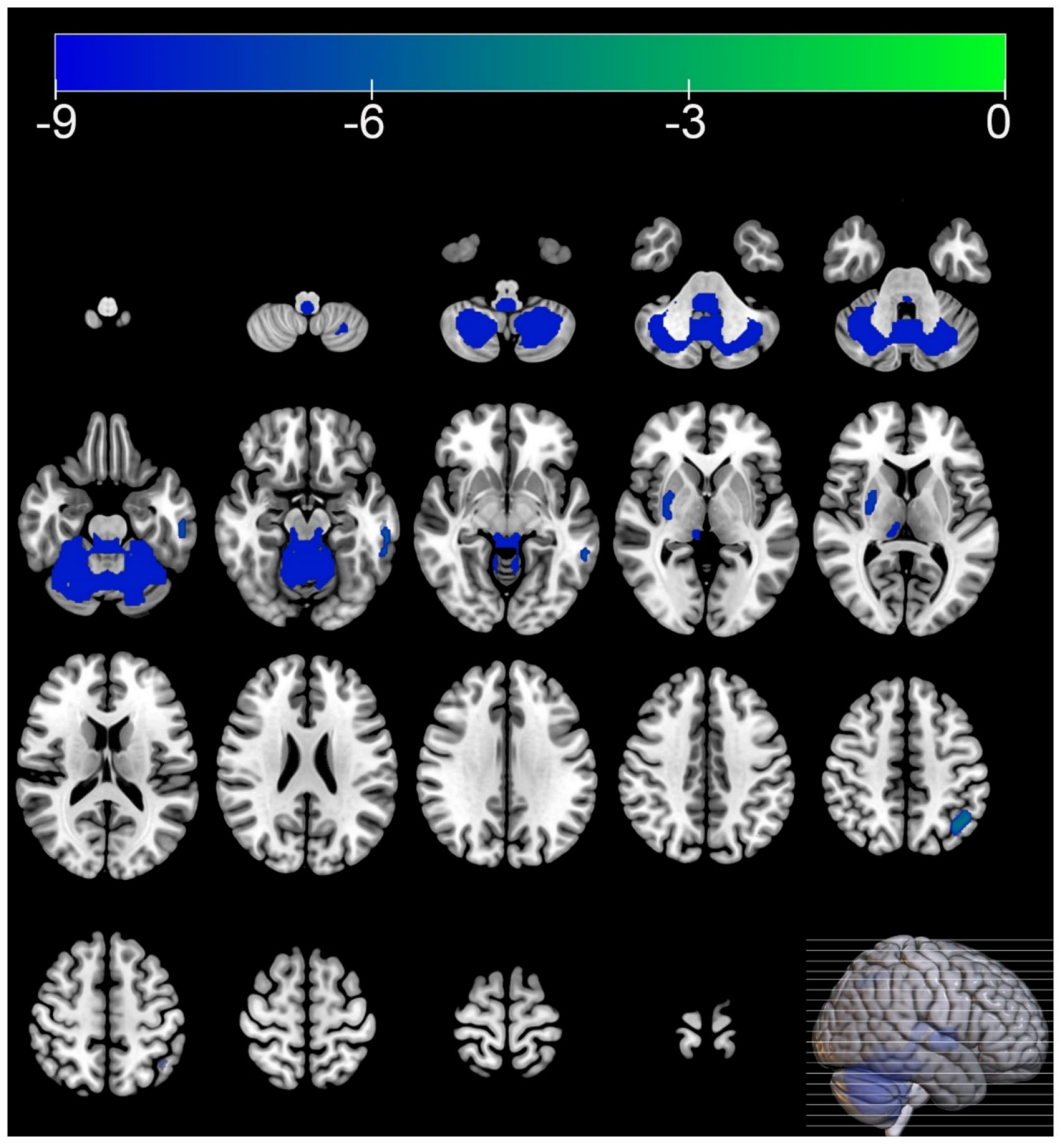
Brain regions with hypometabolism differences between the multiple system atrophy and healthy control groups. The thresholds of the color bars depict the *T*-values.

Compared with the HC group, the MSA group exhibited diffuse reductions in cerebellar glucose metabolism. Additionally, localized reductions in glucose metabolism were observed in the left inferior parietal lobule, right putamen, and left middle temporal gyrus. In contrast, increased glucose metabolism was observed in certain areas of the left postcentral gyrus, right postcentral gyrus, left paracentral lobule (Table 3 and Figures 1, 2).

**Table 3:** Brain regions with glucose metabolism differences between the multiple system atrophy and healthy control groups.

Number	Voxels	Regions	BA	MNI coordinates			*T*-value
				X	Y	Z	
1	9,925	Cerebellum	/	−42	−66	−38	−8.554
2	150	Left inferior temporal gyrus	40	−40	−64	46	−4.882
3	204	Right putamen	/	30	−14	2	−3.762
4	150	Left inferior parietal lobule	20	−60	−26	−18	−4.360
5	611	Left postcentral gyrus	13	−40	−22	28	4.564
6	1,143	Right postcentral gyrus	6	60	−8	26	5.629
7	970	Left paracentral lobule	6	−6	−20	−62	5.903
8	588	Right postcentral gyrus	4	12	−40	70	5.958

A negative *T*-value signifies a decrease in glucose metabolism, and a positive *T*-value signifies an increase in glucose metabolism. MNI, Montreal Neurological Institute; BA, Brodmann area.

### Differences in brain glucose metabolism between the MSA-NC and MSA-CI groups

**Figure 3:**
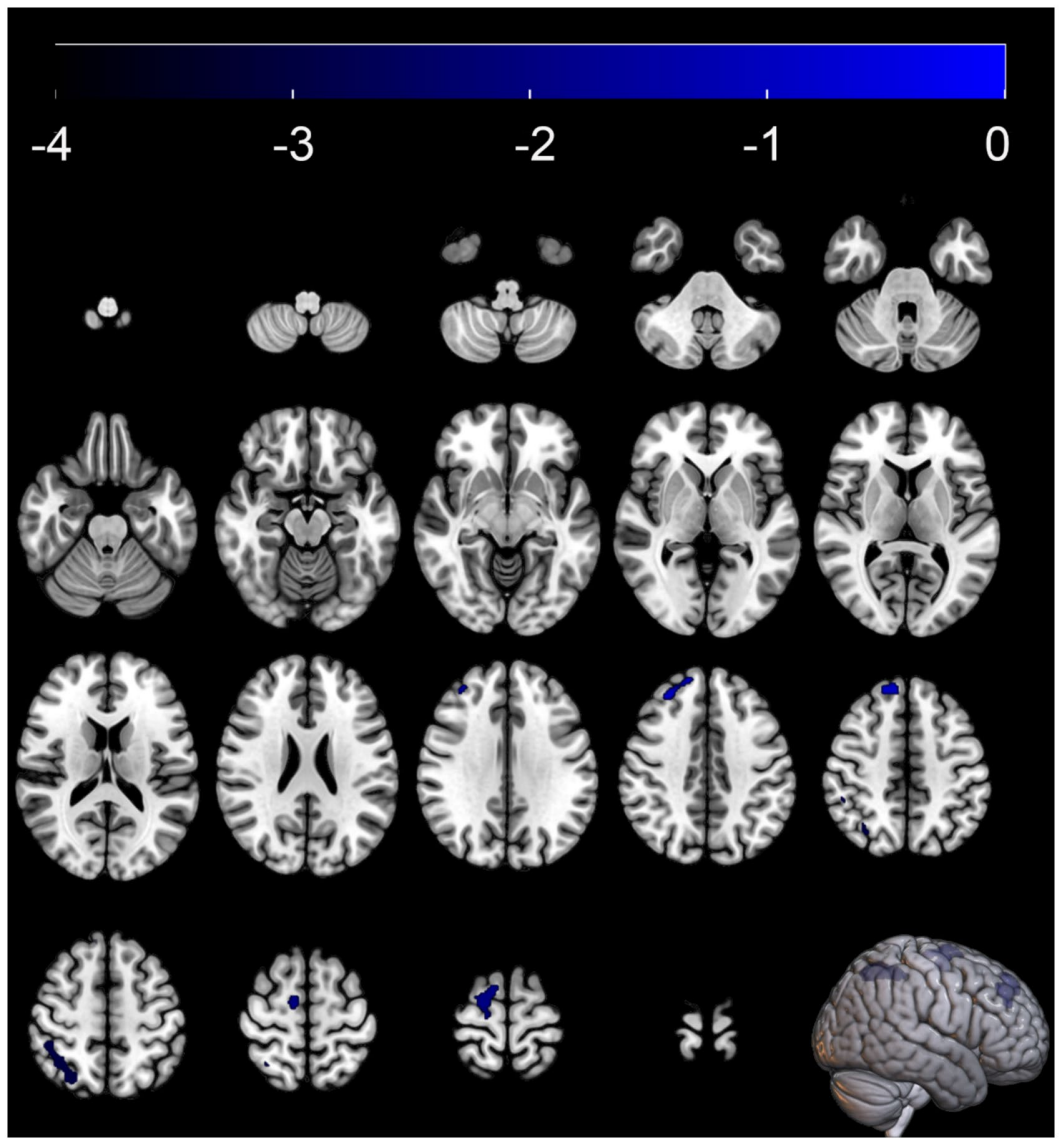
Brain regions with glucose metabolism differences between the multiple system atrophy patients with normal cognition and those with cognitive impairment. The thresholds of the color bars depict the *T*-values.

**Figure 4:**
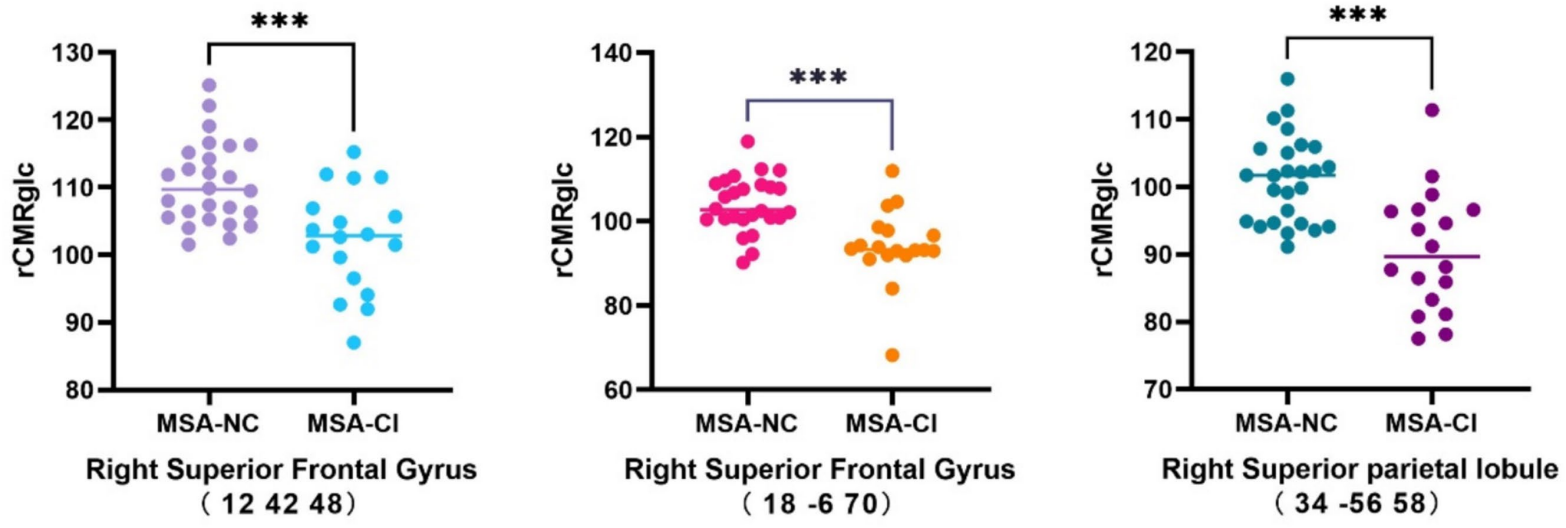
Comparison of the changes in the regional cerebral metabolic rate of glucose in multiple system atrophy patients with different cognitive states. ^***^p < 0.001. MSA-NC, multiple system atrophy with normal cognition; MSA-CI, multiple system atrophy with cognitive impairment; rCMRglc, regional cerebral metabolic rate of glucose.

Compared with the MSA-NC group, the MSA-CI group exhibited reduced glucose metabolism in the right superior frontal gyrus, right superior parietal lobule, and right superior frontal gyrus, with no discernible areas of increased metabolism (Table 4 and Figure 3). The rCMRglc values in the right superior frontal gyrus, right superior parietal lobule, and right superior frontal gyrus regions in the MSA-CI group were 102.31 ± 7.73, 94.10 ± 8.93, and 90.55 ± 8.98, respectively, all of which were significantly lower than those in the MSA-NC group (*p* < 0.001) (Table 5 and Figure 4).

**Table 4:** Brain regions with glucose metabolism differences between the multiple system atrophy patients with normal cognition and those with cognitive impairment.

Number	Voxels	Regions	BA	MNI coordinates			*T*-value
				X	Y	Z	
1	186	Right superior frontal gyrus	9	12	42	48	−3.828
2	328	Right superior parietal lobule	7	34	−56	58	−3.821
3	245	Right superior frontal gyrus	6	18	−6	70	−3.619

A negative *T*-value signifies a decrease in glucose metabolism, and a positive *T*-value signifies an increase in glucose metabolism. (There is no discernible areas of increased metabolism). MNI, Montreal Neurological Institute; BA, Brodmann area.

**Table 5:** Comparison of the regional cerebral metabolic rate of glucose values between the multiple system atrophy patients with normal cognition and those with cognitive impairment.

	Right superior frontal gyrus (12, 42, 48)		Right superior frontal gyrus (18, −6, 70)		Right superior parietal lobule (34, −56, 58)	
	MSA-NC group	MSA-CI group	MSA-NC group	MSA-CI group	MSA-NC group	MSA-CI group
rCMRglc	110.58 ± 6.17	102.31 ± 7.73	104.04 ± 6.52	94.10 ± 8.93	101.05 ± 6.42	90.55 ± 8.98
*p*-value	*p* < 0.001		*p* < 0.001		*p* < 0.001	

The data are presented as means ± standard deviation. MSA-NC, multiple system atrophy with normal cognition; MSA-CI, multiple system atrophy with cognitive impairment; rCMRglc, regional cerebral metabolic rate of glucose.

### ROC curve analysis of brain glucose metabolism between the MSA-NC and MSA-CI groups

**Figure 5:**
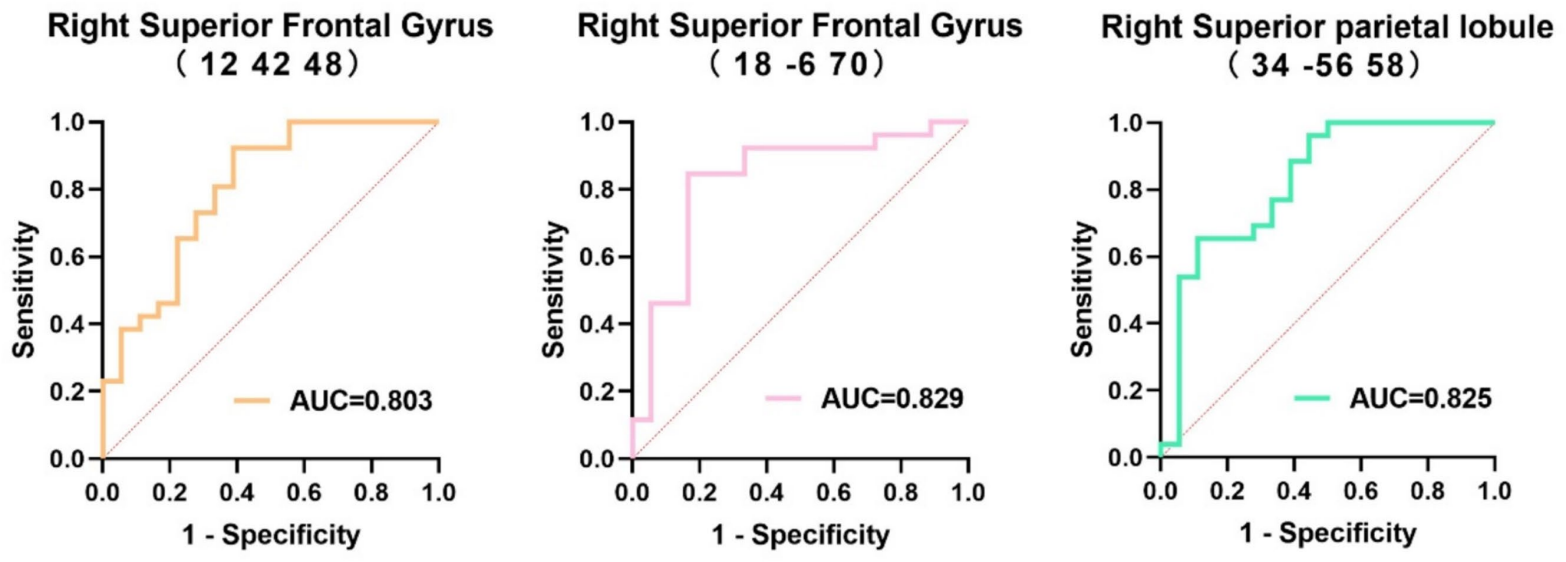
Receiver operating characteristics curve of the regional cerebral metabolic rate of glucose in the three regions to distinguish multiple systemic atrophy patients with different cognitive states. AUC, area under the receiver operating characteristics curve.

The area under the ROC curve (AUC) for the rCMRglc in the right superior frontal gyrus (12, 42, 48), right superior frontal gyrus (18, −6, 70), and right superior parietal lobule (34, −56, 58) to differentiate the MSA patients with cognitive impairment were 0.803 (95%CI = 0.668–0.939), 0.829 (95%CI = 0.696–0.963), and 0.825 (95%CI = 0.693–0.957), respectively, with no statistically significant differences (Figure 5).

The cut-off values for rCMRglc in the right superior frontal gyrus (12, 42, 48), right superior parietal lobule (34, −56, 58), and right superior frontal gyrus (18, −6, 70) to distinguish the cognitive status of MSA patients were 103.86, 99.01, and 99.51, respectively, with corresponding sensitivity and specificity values of 92.3% (95%CI = 75.9–98.6%) and 61.1% (95%CI = 38.6–79.7%), 65.4% (95%CI = 46.2–80.6%) and 88.9% (95%CI = 67.2–98.0%), and 84.6% (95%CI = 66.5–93.9%) and 83.3% (95%CI = 60.8–94.2%), respectively (Table 6).

**Table 6:** The diagnostic efficacy of regional cerebral metabolic rate of glucose in three regions for identifying the cognitive state.

Regions	Optimal cut-off	AUC(95%CI)	Sensitivity	Specificity	Accuracy	Negative predicate value	Positive predicate value
Right superior frontal gyrus (12, 42, 48)	103.86	0.803 (0.668–0.939)	92.3% (75.9%–98.6%)	61.1% (38.6%–79.7%)	79.5%	84.6%	77.4%
Right superior frontal gyrus (18, −6, 70)	99.51	0.829 (0.696–0.963)	84.6% (66.5%–93.9%)	83.3% (60.8%–94.2%)	84.1%	78.9%	88.0%
Right superior parietal lobule (34, −56, 58)	99.01	0.825 (0.693−0.957)	65.4% (46.2%−80.6%)	88.9% (67.2%−98.0%)	75.0%	64.0%	89.5%

## Discussion

MSA is a rare and rapidly progressive neurodegenerative disease, primarily characterized by Parkinsonism, cerebellar impairment, and autonomic dysfunction (Stefanova et al., 2009). The neuropathological hallmark of MSA is the formation of glial cytoplasmic inclusions (GCIs) containing *α*-synuclein (Spillantini et al., 1998). Studies have shown that 20–78% of MSA patients experience varying degrees of cognitive impairment at different stages of the disease, with symptoms such as impaired attention, reduced executive function, memory loss, language disorders, and visuospatial dysfunction. These impairments severely affect the patient’s quality of life (Brown et al., 2010; Kim et al., 2013; Cao et al., 2015; Shen et al., 2021). However, significant variability exists in the reported prevalence of cognitive impairment in MSA across different studies. Previous autopsy studies have suggested that cognitive impairment in MSA may be related to neuronal loss in the frontal cortex and α-synuclein pathology in the limbic regions or the medial temporal lobe (Homma et al., 2016; Koga et al., 2017). However, these pathological findings only represent the end stage of the disease. Therefore, establishing sensitive diagnostic criteria for cognitive impairment in MSA during the clinical stage is crucial.

In this study, 40.9% of the MSA patients were diagnosed with cognitive impairment based on the MMSE, which corroborates previous research findings (Cao et al., 2015; Shen et al., 2021). The SPM analysis of ^18^F-FDG PET images showed that compared with the HC group, the MSA group exhibited reduced glucose metabolism in the cerebellum and putamen. Regional brain metabolism reduction directly correlates with the severity of clinical impairment (Taniwaki et al., 2002). At the same time increased glucose metabolism was observed in certain areas of the frontal and parietal lobes, including the left postcentral gyrus, right postcentral gyrus, left paracentral lobule. Hypermetabolism has been hypothesized as a compensatory mechanism or an early manifestation of neurodegenerative changes (Rubinski et al., 2020; Tondo et al., 2024). These metabolism changes align with the metabolic patterns of brain function associated with MSA reported in previous studies (Eckert et al., 2005).

In the SPM analysis, compared with MSA-NC patients, MSA-CI patients exhibited significantly reduced glucose metabolism in the right superior frontal gyrus and right superior parietal lobule. Several studies have indicated that the prefrontal cortex, as a key hub in the central executive network, is closely related to functions such as memory, cognition, and flexibility. Changes in these brain regions may affect the cognitive status of MSA patients. In a functional MRI study, Li et al. (2023) found that MSA patients with mild cognitive impairment had significantly lower amplitude of low-frequency fluctuations in the right dorsolateral prefrontal cortex compared with MSA-NC patients. Similarly, Yang et al. (2020) discovered that MSA-CI patients had a significantly reduced degree of centrality in the right dorsolateral prefrontal cortex compared with MSA-NC patients, and this reduction correlated negatively with their MMSE scores. From a metabolic perspective, our study further confirms that the right prefrontal cortex is a key region related to cognitive impairment in MSA patients. However, other studies suggest that frontal cortical changes in MSA patients with cognitive impairment are not limited to the right side. A brain perfusion single-photon emission computed tomography imaging study by Kawai et al. (2008) reported that MSA patients with predominant Parkinsonism were more prone to cognitive impairment than those with predominant cerebellar ataxia. Moreover, cognitive and neuropsychological impairments in MSA patients with predominant Parkinsonism were significantly associated with reduced bilateral prefrontal perfusion (Kawai et al., 2008). Additionally, using MRI scans of 72 MSA patients, Fiorenzato et al. (2017) found that MSA-CI patients had smaller left prefrontal cortical volumes than MSA-NC patients. Kaller et al. (2011) noted that during cognitive processes, the left and right prefrontal cortices play different roles. The left prefrontal cortex is primarily involved in the structural analysis of external information and construction of cognitive frameworks, while the right prefrontal cortex is responsible for information processing, integration, and formulation and planning of actions (Kaller et al., 2011). Further studies with larger samples are warranted to confirm these differences and explore the specific mechanisms underlying the cognitive-related hypometabolism in MSA patients.

In addition to observing reduced metabolism in the right frontal lobe of MSA-CI patients, we also noted lower metabolism in the right superior parietal lobule compared with MSA-NC patients. Lyoo et al. (2008) reported that reduced cortical glucose metabolism in MSA patients typically begins in the frontal cortex and gradually spreads to the parietal cortex with disease progression. Previous studies suggested that the superior parietal lobule plays a pivotal role in the regulation of memory, visuospatial abilities, body schema and body image processing, and language skills by interactions between the bilateral frontoparietal networks and the interhemispheric parietal network (Koenigs et al., 2009; Wu et al., 2016; Kumral et al., 2023). A previous autopsy report also indicated that both the frontal and parietal cortices of MSA patients with cognitive impairment exhibited significant atrophy. Under microscopic examination, these atrophic regions showed gliosis and loss of myelinated fibers and axons. Additionally, large amounts of GCIs were observed in the deep layers of the cortex (Konagaya et al., 1999). Those suggested that beyond the frontal lobe, the reduced parietal lobe metabolism would seem to be a key feature of cognitive decline in MSA patients. We did not observe the metabolism changes in cerebellum between the MSA-NC and MSA-CI in our study, which is consistent with the results of the study by Shen et al. (2021). But in another research of Shen et al. (2022), it noted that cerebellum may participated in the pathogenesis of cognitive impairments in MSA patient. This discrepancy may be attributed to the differences in grouping.

The rCMRglc can be used to measure glucose metabolism levels in different brain regions, providing crucial quantitative information about neuronal activity and functional status. In previous studies, rCMRglc has been used for diagnosing dementia, differential diagnosis, and monitoring disease progression (Mosconi et al., 2004; Herholz et al., 2007; Kadir et al., 2012). However, only a few studies have explored the relationship between cognitive function and rCMRglc in MSA patients using voxel-based SPM analysis. In our study, MSA-CI patients had significantly lower rCMRglc values in the right superior frontal gyrus (12, 42, 48), right superior frontal gyrus (18, −6, 70), and right superior parietal lobule (34, −56, 58) compared with MSA-NC patients. The ROC analysis of rCMRglc in these regions showed that the rCMRglc in the right superior frontal gyrus (18, −6, 70) had a high diagnostic value for distinguishing between MSA-NC and MSA-CI patients, with a sensitivity and specificity of 84.6 and 83.3%, respectively. Using standardized data processing and analysis methods to examine the ^18^F-FDG PET images of patients, we could minimize the impact of human error on the measurements, yielding results with better stability and reproducibility. This approach effectively addresses the limitations of the MMSE, which requires a high level of clinical expertise and is susceptible to subjective influences. Therefore, this method holds promise as an effective tool for assisting in the clinical diagnosis of cognitive impairment in MSA patients.

This study has certain limitations. MMSE is a preliminary screening tool for assessing overall cognitive function, but its limitation lies in the inability to comprehensively evaluate cognitive performance across multiple domains. When combined with more effective cognitive assessment tools, the additional value of PET scans in cognitive status evaluation may be reduced. What’s more, owing to the relatively limited sample size, we did not detail stratification of MSA patients based on distinct clinical subtypes and varying cognitive levels and domains, nor did we compare glucose metabolic differences among these subgroups. Future research should aim to incorporate a larger cohort and conduct more comprehensive analyses of patient clinical characteristics to enhance the generalizability of the findings and bolster the robustness of the conclusions.

## Conclusion

In ^18^F-FDG PET imaging, the glucose metabolism characteristics of MSA patients are characterized by reduced glucose metabolism in the putamen and cerebellum and increased glucose metabolism in the bilateral frontal and parietal cortices. Compared with the MSA-NC group, the MSA-CI group exhibits significantly reduced glucose metabolism in the right superior frontal gyrus and superior parietal lobule. The rCMRglc value of the right superior frontal gyrus (18, −6, 70) shows potential as a molecular imaging biomarker for evaluating cognitive impairment in MSA patients.

## Data Availability

The original contributions presented in the study are included in the article/supplementary material, further inquiries can be directed to the corresponding authors.

## Footnotes

^1^ https://www.nitrc.org/projects/mricron

^2^ http://www.fil.ion.ucl.ac.uk/spm/

^3^ http://marsbar.sourceforge.net
